# Effectiveness and feasibility of a software tool to help patients communicate with doctors about problems they face with their medication regimen (EMPATHy): study protocol for a randomized controlled trial

**DOI:** 10.1186/s13063-015-0672-7

**Published:** 2015-04-10

**Authors:** John Billimek, Herlinda Guzman, Marco A Angulo

**Affiliations:** Health Policy Research Institute and Division of General Internal Medicine, School of Medicine, University of California, Irvine, 100 Theory, Suite 110, Irvine, CA 92697 USA; Department of Family Medicine, School of Medicine, University of California, Irvine, 101 The City Drive South, Building 200, Suite 835, Orange, CA 92868 USA

**Keywords:** Type 2 diabetes, Medication adherence, Computer-assisted interventions, Doctor-patient communication, Health disparities

## Abstract

**Background:**

Low-income, Mexican-American patients with diabetes exhibit high rates of medication nonadherence, poor blood sugar control and serious complications, and often have difficulty communicating their concerns about the medication regimen to physicians. Interventions led by community health workers, non-professional community members who are trained to work with patients to improve engagement and communication during the medical visit, have had mixed success in improving outcomes. The primary objective of this project is to pilot test a prototype software toolkit called “EMPATHy” that a community health worker can administer to help patients identify the most important barriers to adherence that they face and discuss these barriers with their doctor.

**Methods/Design:**

The EMPATHy toolkit will be piloted in an ongoing intervention (Coached Care) in which community health workers are trained to be “coaches” to meet with patients before the medical visit and help them prepare a list of important questions for the doctor. A total of 190 Mexican-American patients with poorly controlled type 2 diabetes will be recruited from December 2014 through June 2015 and will be randomly assigned to complete either a single Coached Care intervention visit with no software tools or a Coached Care visit incorporating the EMPATHy software toolkit. The primary endpoints are (1) the development of a “contextualized plan of care” (i.e., a plan of care that addresses a barrier to medication adherence in the patient’s daily life) with the doctor, determined from an audio recording of the medical visit, and (2) attainment of a concrete behavioral goal set during the intervention session, assessed in a 2-week follow-up phone call to the patient. The statistical analysis will include logistic regression models and is powered to detect a 50% increase in the primary endpoints.

**Discussion:**

The study will provide evidence regarding the effectiveness and feasibility of a software tool to help patients communicate with doctors about problems they face with their medications.

**Trial registration:**

ClinicalTrials.gov NCT02324036 Registered 16 December 2014.

## Background

Although numerous effective and affordable medication therapies for diabetes exist, and others are being developed, the true impact on health of the best available medications is greatly limited by widespread medication nonadherence [1,2]. Even as access to health care continues to improve nationwide, only 19% of diabetic patients have attained recommended targets for glycemic control, lipid levels and blood pressure [3], and around 50% struggle to adhere to their medications [2,4]. This is especially problematic for low-income, ethnic minority patients who face challenging life circumstances that make adherence difficult [5-8] and who, not surprisingly, suffer the worst health outcomes [9,10]. Even those who gain access to health insurance often do not realize improved outcomes [11]. This can be explained largely by poor medication adherence [10] and a lack of health care that is responsive to barriers in patients’ daily lives [12].

Nonadherence is a highly heterogeneous set of behaviors [13], and the reasons people deviate from their regimens vary considerably [14]. Reasons for nonadherence include high out-of-pocket costs, forgetting to take the medication, difficulties obtaining refills, concerns about side effects, doubts about the necessity of the medication and other unfavorable beliefs about taking the medication [5,14,15]. Numerous interventions and resources to help promote adherence, such as pharmacist-led medication management clinics and cell phone-based reminders, are available [16,17], but to have maximum effect, the right intervention should be matched to the specific barriers an individual is facing [13].

It is no surprise, therefore, that effective doctor-patient communication is associated with better regimen adherence [18]. It leads to patients who are more engaged in the medical decision-making process and are therefore more likely to follow through with their regimen and attain better clinical outcomes [19,20]. Furthermore, it allows the patient to engage in a discussion of his or her personal life circumstances and allows the physician to tailor the regimen to a specific patient’s circumstances [21-23], which has been shown to promote improved outcomes [24].

Despite these benefits, and recent guidelines recommending that providers consider patient’s life circumstances when developing a treatment plan for diabetes [25], there is often not enough time in a 15-minute visit to discuss all the possible barriers to adherence. On average, patients discuss six health issues with their doctor in each visit [26], but only address the non-medical, “contextual” barriers to adherence (such as costs, confusion and health beliefs) in 8% of visits [27]. For many patients, it is difficult to communicate with providers about barriers [28], especially when there are language and cultural differences [10]. Unfortunately, these visits are short and busy [26], and patients—especially those from disadvantaged populations—often do not communicate effectively with providers about their specific barriers to medication adherence [10,28].

Empowering patients to prepare in advance of their visit to identify their most important barriers to adherence may promote a focused discussion of those barriers that is feasible during a short, busy medical visit. Community health worker (CHW)-led interventions show promise to improve adherence and outcomes by promoting better patient-provider communication, but to date have produced mixed results [29]. CHWs are “non-clinical” members of a health care team who come from similar backgrounds in terms of ethnicity, language, socioeconomic status and life experiences as the patients they serve [30]. CHWs have been employed widely to provide culturally appropriate health education in diverse settings [31]. Although well-trained CHWs exhibit excellent cultural competency, identifying the most important issues to address in a complex regimen remains a challenge for providers at all levels of training [32]. CHW-led interventions maybe improved by introducing a component to structure the discussion and prioritization of barriers to medication adherence.

A computer-assisted approach may improve the ability of patients and CHWs to work together to identify and prioritize key barriers to adherence prior to the medical visit. Such approaches can be designed to be inexpensive and to require minimal additional training, while making better use of the patient’s wait time prior to the doctor visit [33].

We hypothesize that a computer-assisted intervention combining a software toolkit to guide the discussion of barriers to medication adherence with an existing CHW-led patient participation training intervention [34] will help patients communicate effectively with doctors about problems they face with their medication regimens. In this article, we describe this software tool, the EMPATHy Toolkit, and the protocol for a randomized controlled trial to assess the feasibility and effectiveness of the tool.

### Objectives

The primary objective of this project is to pilot test “EMPATHy,” a prototype software-based toolkit in a previously developed CHW-led intervention (Coached Care) that is ongoing at the study site with a highly vulnerable population—low-income Mexican-American patients with diabetes and a history of nonadherence. Results from this pilot study will be used to estimate the key parameters necessary to design a definitive trial and ascertain the feasibility and usability of the EMPATHy toolkit.

All study comparisons will be between patients randomly allocated to a “Coached Care + EMPATHy” group versus those assigned to a control group (“Routine Coached Care” with no software).

The specific aims of the project are:

- *Aim 1.* Evaluate the preliminary impact—after a single intervention visit—of EMPATHy on doctor-patient communication about problems patients are facing with their medication regimens. We hypothesize that compared to controls, patients in the “Coached Care + EMPATHy” condition will:
  1.1 Be more likely to raise a relevant “contextual factor” (i.e., a barrier to medication adherence in daily life) with the doctor, determined using a validated audio coding scheme [35] to analyze an audio recording of the medical visit.
  1.2 Be more likely to leave the medical visit with a “contextualized plan of care” (i.e., a plan of care that addresses the contextual factor raised during the visit), determined using the same audio coding scheme.
- *Aim 2.* Evaluate the impact of EMPATHy on patient follow-through with the plan of care developed during the medical visit with the doctor. We hypothesize that compared to controls, patients in the “Coached Care + EMPATHy” condition will:
  2.1 At 2-week follow-up, be more likely to have attained a concrete behavioral goal identified during the intervention visit.
  2.2 By the next regularly scheduled medical visit, be more likely to show improvement in the “red flag” health outcome (A1c, LDL or blood pressure) identified during the intervention visit.
- *Aim 3.* Assess and describe the feasibility, acceptability and usability of EMPATHy.

## Methods/Design

### Study design

This study is a randomized controlled trial allocating participants to complete either the standard Coached Care intervention (routine coached care intervention with no software enhancement and unstructured discussion of barriers) or enhanced Coached Care (the Coached Care + EMPATHy software to structure discussion of barriers). Standard Coached Care is a program offered to eligible patients with diabetes at the study site and therefore is considered routine care.

### Study setting

Participants will be recruited from a primary care clinic in a university-affiliated federally qualified health center in Santa Ana, California. The center predominantly serves low-income, ethnic minority patients from the community.

### Interventions

The EMPATHy toolkit will be pilot tested in the context of a previously developed CHW-led intervention known as Coached Care. The Coached Care intervention is a CHW-led patient participation training intervention developed and tested in a prior study [34]. Patients in the program are paired with CHW “coaches,” matched to the patient’s language and ethnicity. The coaches meet with patients immediately prior to their medical visits to discuss barriers the patient is facing. The objective of a coaching session is to help the patient develop helpful questions to ask the physician about specific barriers and to provide training to help the patient ask the questions confidently and effectively.

Coaches are recruited through job postings posted on the medical center “Careers” website and in advertisements posted throughout the clinic. Coaches are not required to have formal education beyond a high school diploma or equivalent or to have experience working in a health care setting. For coaches recruited to work at sites with many patients who speak a language other than English, bilingual proficiency in that language is required. “A personal connection” to a chronic health condition is listed as a preferred qualification.

Newly hired coaches complete 4 days of standard “on-boarding” training required of all clinic employees on topics including confidentiality, safety, data security compliance and using the electronic medical record and other computer systems. They then complete online training modules in motivational interviewing, “teach-back” methods for patient education and an introduction to diabetes and heart failure. Next, they complete 2 days of classroom training discussing the etiology, symptoms and management of the chronic conditions that will be the focus of their work (diabetes and heart failure) and the principles of patient participation training (discussing barriers to disease management, formulating questions for the doctor, coaching the patient to discuss their questions effectively, etc.). They then complete 2 weeks of on-site training in the clinics with the intervention coordinators and have continuing education activities at least once per quarter.

#### Study intervention (Coached Care + EMPATHy software)

**Figure 1:**
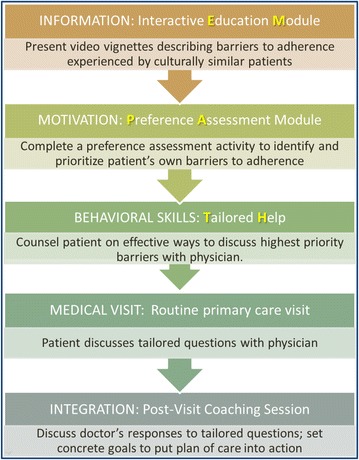
**Conceptual overview of the EMPATHy Toolkit.**

Participants allocated to the study intervention will complete a Coached Care intervention visit enhanced by completing a tablet computer-based activity using the EMPATHy toolkit. The conceptual model for EMPATHy is grounded in the Information-Motivation-Behavioral Skills (IMBS) model of medication adherence [36], which suggests that for a patient to adhere to a medication regimen, he or she requires (1) information—an understanding of what needs to be done, (2) motivation—a belief that adherence will produce a worthwhile benefit and (3) behavioral skills—strategies and resources to overcome barriers to adherence (see Figure 1).

The toolkit, therefore, consists of three components: (1) a computer-based interactive Educational Module (EM) to convey information about the complex set of benefits and burdens associated with a disease management regimen; (2) a Preference Assessment (PA) module to help the patient identify and prioritize the most important barriers that outweigh the perceived benefits of taking medications; and (3) a Tailored Help (TH) module using the results from the PA to guide the coach to teach behavioral skills tailored to help patients overcome those high priority barriers.

**Figure 2:**
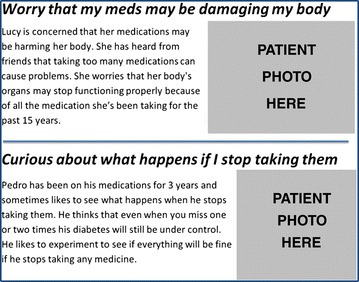
**Sample vignettes from the Education Module (EM).**

**Figure 3:**
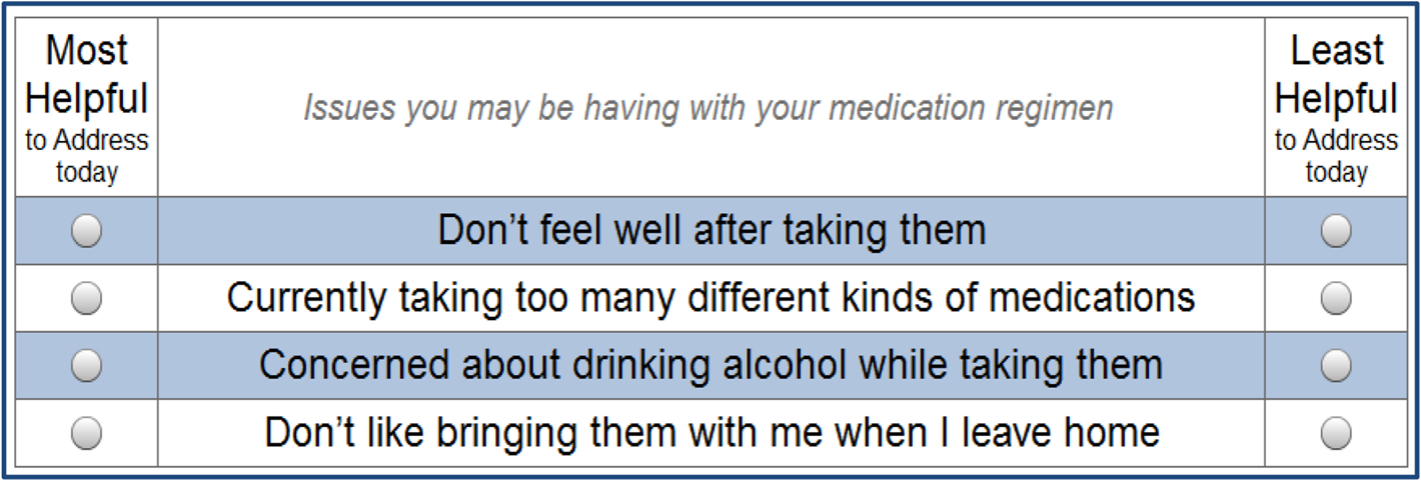
**Sample screenshot, in English, of a page in the Preference Assessment (PA) module.**

1. Education Module (EM). The EM consists of a set of vignettes describing each of a set of commonly experienced barriers to medication adherence. The vignettes present each barrier in practical terms as a real person’s experience of a barrier (e.g., describing how a person might be affected by concerns about costs or doubts about the safety of the drug; see Figure 2 for example).
  Broad themes for barriers to include in the Education Module materials were identified from a literature review and interviews with patients, physicians, diabetes educators and other experts, then vetted by an advisory group of patient stakeholders. For each barrier, a brief educational page was written at a sixth grade reading level and translated from English to Spanish by a team of natively fluent, trained translators. For this prototype tool, the EM materials are presented as a series of simple web pages in Spanish and English with navigation buttons to move from topic to topic in any order selected by the patient. Navigation through the topics is driven by the patient and is facilitated by the coach who is trained in the use of the EM.

2. Preference Assessment (PA) module. The PA module was developed using a commercially available preference assessment software package (SSI Web v.8.3, Sawtooth Software, Orem, UT). The PA module applies a preference assessment method known as maximum difference scaling (MDS or “best-worst” scaling), guided by best practices for stated-preference assessment methods for conjoint analysis and discrete choice experimental methods [37]. Sawtooth Software allows MDS tasks to be run and analyzed on desktop and mobile devices with no specialized programming knowledge and has been successfully used in many settings, including in studies of patients with very low socioeconomic status [38]. The PA has been tailored to the appropriate level of literacy, numeracy, health literacy and graphical literacy for the population we work with [39,40].
  MDS methods are useful to identify the “most important” elements of a decision or situation for an individual [41,42]. They are less cognitively demanding than most preference assessment tasks (such as rank ordering a long list of options), requiring the respondent to consider just a few options at a time. They are less vulnerable to certain biases than simple tasks such as “pick one” tasks (e.g., “Name the most important barrier to taking your medications”). Finally, they differentiate the importance of individual options better than asking a respondent to rate their importance on a numeric scale (e.g., when someone is asked to “rate on a 1 to 10 scale how important each of these things are to you,” they tend to rate several things as very important, but do not identify the most important thing). MDS, on the other hand, is an excellent approach to help individuals identify the one or two most important barriers to discuss with the doctor because it allows them to consider an array of possible barriers head to head against each other, a few barriers at a time, several times each, to see which issues “rise to the top” as the most important [41].
  Low socioeconomic status patients have a difficult time identifying the most important topics to discuss with the doctor and often end up not discussing any barriers to adherence during the visit. Even if the task does not identify the true “most important barrier” for a given patient, we posit that the activity will at least start a discussion about barriers with the doctor that will encourage developing a plan of care that addresses challenging life circumstances.
  The MDS task created for the Preference Assessment (PA) module includes the barriers described in the EM. The MDS task presents four of these barriers at a time, in random order, and asks the patient to indicate which barrier would be the most helpful to discuss during the visit, and which would be least helpful. A total of nine pages with new combinations of four barriers are presented until one or two high-priority barriers are identified (see Figure 3 for a sample page).

3. Tailored Help (TH). The ultimate intended use of the EMPATHy toolkit is as an application that can be integrated with the protocol of any CHW-led intervention to provide “tailored help” to the patient. Intervention coordinators would first identify which elements of their intervention (resources, programs, educational materials, etc.) would be most helpful to address each type of barrier included in the EM and PA modules. These “best options” for each barrier type would be noted in the software. Then, in intervention visits, the CHWs would use the toolkit to assess the high-priority barriers for each patient and then provide a tailored response consisting of the “best option” intervention elements to address that barrier.
  For this project, we will test the effectiveness of incorporating EMPATHy with the Coached Care intervention. In the Coached Care intervention, the main product of each coaching encounter is a tailored list of questions for the physician about the barriers to adherence faced by the patient. The TH module for this prototype will consist of a set of recommended questions for the physician that match each barrier evaluated in the PA. During the intervention session, the CHW coach will refer to this set as he or she helps the patient come up with a list of questions to address the patient’s high priority barriers.

#### Active comparator (standard coached care)

Participants allocated to the active comparator will complete a standard Coached Care intervention visit with no computer-based activity. Instead, the patient and coach will have an unstructured discussion of barriers to adherence. Additional details on the content of intervention visits for both study conditions can be found under “Study Procedures.”

### Study procedures

**Figure 4:**
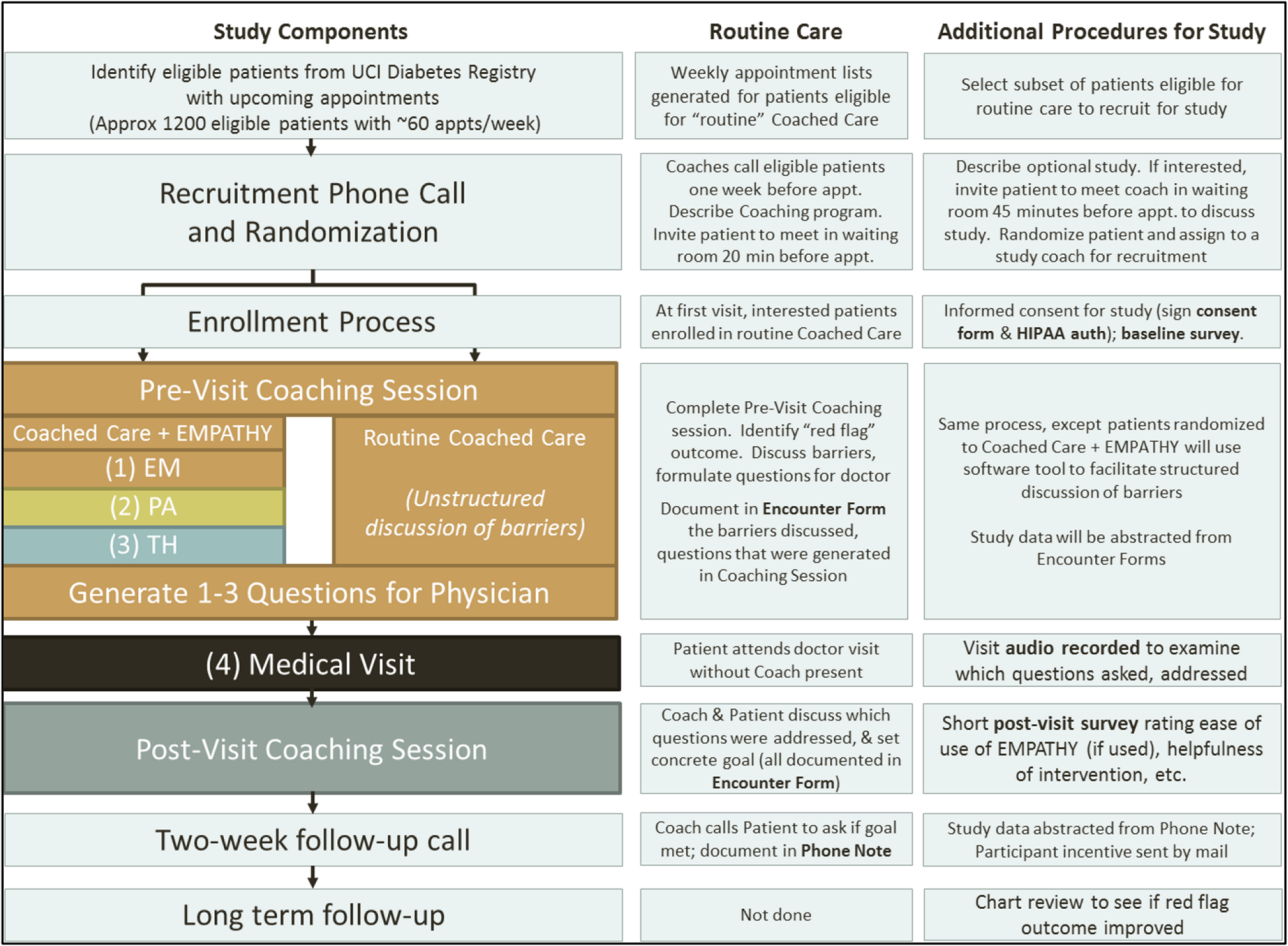
**Protocol summary delineating routine care activities (for any patient enrolled in Coached Care at the clinics) versus additional procedures unique to the study.**

The study protocol, delineating routine care activities (for any patient enrolled in Coached Care at the clinics) versus additional procedures unique to the study, is summarized in Figure 4. The augmented Coached Care intervention using the EMPATHy toolkit will be pilot tested in a sample of patients currently eligible to participate in the UC Irvine Health Coached Care Intervention. Two “study coaches,” in addition to the coaches providing usual care, were hired to conduct all study activities and were trained in the Coached Care intervention, use of the EMPATHy toolkit and all study procedures. Both study coaches are bilingual in English and Spanish and have a bachelor’s degree from a university. Patients enrolled in this pilot study will be randomly assigned either to complete a single coaching session using EMPATHy or to complete a single coaching session with no computer-assisted activity. Both coaches deliver both versions of the intervention. Components of the intervention visit and follow-up activities are described below.

#### Sample identification

Participants will be identified from the study site’s diabetes registry, which includes all adult patients actively receiving treatment for diabetes at the study site.

#### Recruitment phone call

The coach will telephone eligible patients who have a scheduled medical appointment at the study clinic in the next 21 days. For the study, patients will be asked to meet the coach at the clinic 45 minutes before the scheduled appointment to discuss the study, give informed consent if interested and complete the pre-visit coaching session.

#### Study enrollment

Interested prospective participants will meet with the study coach in a private area in the clinic to discuss the study and, if interested, sign a consent form and HIPAA authorization form. Once informed consent is granted, the participant will be asked to complete a brief baseline survey, available in English or Spanish, with questions about sociodemographic characteristics, barriers to disease management and medication adherence. Participants will be assured that they can opt not to respond to any of the questions. The coach can read questions to any participants having difficulty reading the survey.

#### Pre-visit coaching session

All study participants will complete an intervention visit based on the routine Coached Care program in which patients are paired with a CHW coach, matched to the patient’s language and ethnicity. In both study conditions, the coach and patient will review the medical record from their most recent visit to identify one or more “red flag” issues related to their disease management such as high hemoglobin A1c, cholesterol, etc. Patients randomized to the active comparator (Coached Care with no software tools) will have an unstructured discussion of barriers to chronic disease management that may be contributing to the red flag outcome. Participants randomized to the study intervention (Coached Care + EMPATHy) will work with the coach to complete the computer-assisted activity using a tablet computer. The computer-based activity, described in more detail under “Study Intervention,” includes a brief Educational Module (EM) reviewing vignettes of barriers that many patients face, followed by a Preference Assessment (PA) module to obtain a rank ordering of the highest priority barriers that interfere with the patient’s adherence to their medication regimen and then Tailor Help (TH) to focus the rest of the visit on the highest priority barriers. In both conditions, the coach will then employ the original Coached Care protocol to help the patient generate one to three questions for the physician focusing on the highest priority barriers and will note these questions in the Encounter Form.

#### Medical visit

After the intervention visit, the patient will bring the list of questions into their regularly scheduled medical visit. With permission of the patient and the physician, the visit will be audio recorded using a portable digital recorder that the coach will place in the examination room and collect after the visit. Before beginning the recording, the coach will remind the patient that recording the visit is optional. If either the patient or the physician prefers that the visit not be recorded, the coach will not record the visit. In cases where the recording was not done, the study endpoints that require audio recordings will not be evaluated for that patient, but the remaining endpoints will be evaluated from the other data sources. No members of the study team will attend the medical visit, but will wait until after the visit to collect the recorder.

#### Post-visit session

After the medical visit, the patients in both study conditions will meet with the coach to review the list of questions from the pre-visit coaching session. They will discuss whether the patient was able to get an answer to each of the questions and discuss the answers provided. The coach and patient will then create a plan with specific actions the patient should take in response to the answers provided by the doctor. The coach will note in the Encounter Form which questions were asked, which were answered and what action steps were recommended. The patient and coach will then set a single, simple concrete behavioral goal for the patient to complete in the subsequent 2-week period. This goal will also be noted in the Encounter Form. Finally, the patient will complete a brief post-visit questionnaire about the ease of use of the EMPATHy software (if they used it), the helpfulness of the coaching session, the relevance of the issues and concrete goals identified in the coaching session, and confidence that his or her outcomes will improve. Immediately upon completing the post-visit session, the coach will hand the participant a $20 gift card for compensation.

#### Two-week follow-up call

Two weeks after the visit, the coach will call each participant to review the concrete goal and will document in the Phone Note whether or not the patient completed the goal. They will discuss any barriers to completing the action plan and will encourage the patient to follow through. Finally, primary nonadherence will be assessed by asking whether the patient filled all prescriptions written during the study medical visit. After completing this phone call, the participant will be given a second $20 gift card for compensation.

#### Long-term follow-up

Six months after the intervention visit, the patient’s medical record will be reviewed to obtain the patient’s most recent laboratory values to examine whether the “red flag” outcome identified in the pre-visit session improved since the coaching session.

### Ethics

The research protocol has been approved by the University of California Irvine Institutional Review Board (HS#2014-1441).

### Inclusion criteria for patients

Patients will be recruited from the UC Irvine Federally Qualified Health Center family medicine clinic sites in Santa Ana and Anaheim with the following inclusion criteria: (1) age 18 and older; (2) have poorly controlled type 2 diabetes (as indicated by HbA1c >7.5%, LDL cholesterol >100 mg/dl or systolic blood pressure >140), (3) be of Hispanic ethnicity and (4) speak English or Spanish.

### Outcome assessments

**Figure 5:**
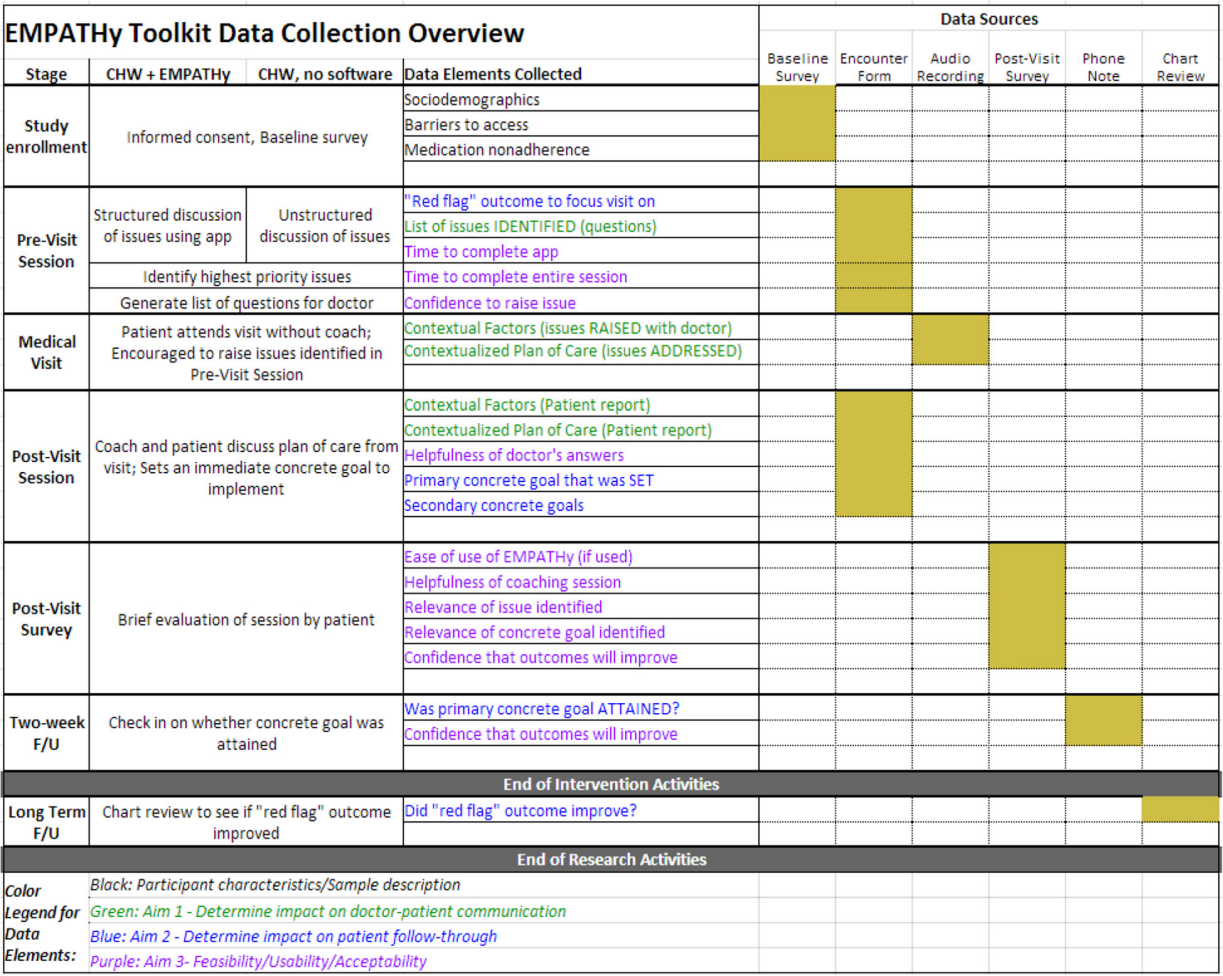
**Data collection overview.**

Study endpoints will be assessed from (1) analysis of audio recordings of the patient’s medical visit with the doctor, (2) the Encounter Form used to document the topics discussed in the Pre- and Post-Visit Coaching Sessions, (3) a brief 2-week follow-up phone call documented in a “Phone Note,” (4) a brief Post-Visit survey and (5) abstraction of laboratory and blood pressure data (HbA1c, LDL cholesterol and blood pressure) from the electronic medical record at 6-month follow-up. Participant characteristics, barriers to access and baseline medication nonadherence will also be assessed with (6) a brief baseline survey. Study outcome assessments are summarized in Figure 5.

*Primary Outcomes.* Two primary outcomes will be evaluated for this study.

1. Development of a contextualized plan of care will be assessed by analyzing an audio recording of the medical visit using a previously developed coding scheme [35] (see “Analysis of Audio Recordings”). If the coder concludes that the plan of care discussed in the visit adequately addresses a contextual factor raised during the visit, it will be determined that a contextualized plan of care was developed.
2. Attainment of a concrete behavioral goal will be assessed in a 2-week follow-up phone call to the patient and will be defined as the patient reporting having completed the specific action that he or she specified with the Coach as a concrete goal during the intervention post-visit.

*Secondary Outcomes:* Two secondary outcomes will be assessed.

1. Discussion of a contextual factor in the visit will be assessed from audio recordings of the visit using a previously developed coding scheme [35]. If the coder concludes that a relevant barrier to the patient taking his or her medication consistently was raised during the visit, it will be determined that a contextual factor was discussed.
2. Improvement in “red flag” outcomes will be assessed from the patient’s medical record as the change in the measured value of the outcome measure identified as a high priority outcome (“red flag”) from the intervention visit date till the next regularly scheduled assessment. The red flag outcome measure is selected by the Coach and patient during the intervention visit and can be either hemoglobin A1c, LDL cholesterol or blood pressure level.

#### Additional measures

Several measures of feasibility, acceptability and usability will be collected to describe the participants’ experience with the software tools and the intervention. The measures include: the time required to complete the intervention sessions, the patients’ ratings of the ease of use of the EMPATHy toolkit, confidence to raise the contextual factor with the doctor, relevance of contextual factors discussed, the helpfulness of the doctor’s responses, the relevance of the concrete goals during the intervention visit and confidence that outcomes will improve.

Patient-reported medication adherence will also be assessed using an 11-item self-report measure of patients’ deviations from the prescribed regimen in the face of specific barriers [15,34,43]. This measure has been used in this patient population, is highly correlated with blood sugar control and LDL cholesterol, and captures both the extent of nonadherence and reasons for nonadherence. Additionally, primary nonadherence (failure to fill a prescription) will be collected from patient report during the 2-week follow-up call.

#### Analysis of audio recordings

**Figure 6:**
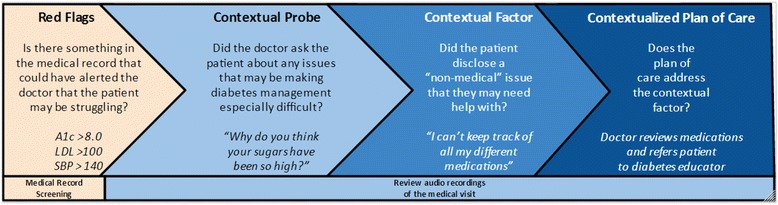
**Overview of coding method, Content Coding for Contextualization of Care (4C).**

Audio recordings of the medical visits will be coded by members of the research team using a validated method known as Content Coding for Contextualization of Care (4C) [35,44] (see Figure 6). The coding method involves identifying (1) “red flags” or health issues that warrant discussion of barriers to disease management, (2) whether a “probe” from the doctor asking about barriers that may have contributed to the red flag occurred, (3) whether a “contextual factor” or specific barrier was raised by the patients, and (4) whether a “contextualized plan of care” was created to address the barrier raised. Inter-rater reliability will be evaluated by having each recording coded by two separate coders, and then any disagreement between the raters will be resolved by the lead researcher. Prior studies using the 4C method have reliably identified how frequently contextual factors are discussed [27] and shown that patients who received a contextualized plan of care experience improved outcomes compared to those did not [24].

### Statistical analysis

Participant characteristics, including age, gender, education, type of medical insurance, nativity and employment status, will be compared between groups using univariate statistics. Primary and secondary outcomes are all dichotomous (yes or no) outcomes and will be compared between groups using logistic regression models. Feasibility, usability and patient-reported barriers to nonadherence will be presented descriptively.

#### Sample size

The target sample size is 190 (95 participants per group) to allow us to detect a 50% difference in the rate of occurrence of the primary outcome between the intervention and comparison group with 80% power and an alpha = 0.05. This assumes we observe a base rate of the outcome of 40% in the active comparator group, as we have observed in an unpublished analysis of data from a prior study, and 60% in the EMPATHy + Coached Care intervention group.

The sample size calculations do not account for loss to follow-up because the first primary outcome (development of a contextualized plan of care) is collected at the single intervention visit, and the second primary outcome (attainment of a concrete behavioral goal) is collected via telephone after a fairly short follow-up period (2 weeks) in patients who regularly access the clinic. Results from this pilot study will be used to estimate the rate of loss to follow-up for a definitive effectiveness trial.

## Discussion

Numerous studies have employed preference assessment methods such as conjoint analysis and maximum difference scaling (MDS) to examine patient preferences about attributes of available treatment options to help guide treatment choice. The innovation of the current study is to apply similar methods not to help select a medication that is compatible with a patient’s preferences, but to elicit a discussion of barriers to following the regimen the patient has already been prescribed.

This is important because a large share of disadvantaged patients struggle to adhere to their medication regimens, and it is for reasons that extend beyond out-of-pocket costs or forgetting to take their pills [5,15]. Although many patients benefit from programs such as pharmacist-led medication management clinics and cell phone-based reminders [17], these interventions cannot be applied unless nonadherence is identified [13]. For many patients, the only opportunity for the health system to identify and respond to nonadherence is during the medical visit with the physician. Unfortunately, these visits are short and busy [26], and patients—especially those from disadvantaged populations—often do not communicate effectively with providers about their specific barriers to medication adherence [10,28]. By helping patients prepare to discuss their highest priority barriers to adherence prior to the visit, approaches like the EMPATHy toolkit may improve the ability of the health system to recognize nonadherence and its underlying reasons and to match patients up with resources and interventions to overcome barriers to adherence.

Due to its limited scale as a pilot project, the current study has a number of limitations. First, the study focuses solely on medication adherence, but not on other important diabetes management behaviors such as diet, physical activity and self-monitoring of blood glucose. Second, the study relies on a single intervention visit to impact the discussion of barriers to adherence, which may be inadequate for many patients to give sufficient consideration to their barriers to identify the most important ones to discuss, or to feel comfortable discussing barriers at all with a CHW that they have met only once. Third, there is no objective assessment of medication adherence using methods such as medication electronic monitoring system (MEMS) pill bottle caps that record each time a pill bottle is opened to take a medication. These limitations can be addressed in larger scale studies evaluating interventions with a broader focus, administered over multiple visits, with objective follow-up measures of adherence. The present study, however, will provide valuable pilot data to estimate key parameters to design a definitive trial to test the effectiveness of approaches like the EMPATHy toolkit.

### Trial status

The randomized trial is currently in the phase of participant enrollment and follow-up.

## Abbreviations

CHW: Community health workers
EMPATHy: (EM) Educational Module
(PA): Preference assessment
(TH): Tailored help
IMBS: Information-motivation-behavioral skills
MDS: Maximum difference scaling
MEMS: Medication electronic monitoring systems
